# Does the implementation of pay-for-performance indicators improve the quality of healthcare? First results in France

**DOI:** 10.3389/fpubh.2023.1063806

**Published:** 2023-03-09

**Authors:** Marc-Antoine Sanchez, Stéphane Sanchez, Leila Bouazzi, Louise Peillard, Aline Ohl-Hurtaud, Catherine Quantin

**Affiliations:** ^1^Information Systems and Digital Department, French Military Health Service, Saint-Mandé, France; ^2^Centre de recherche en Epidémiologie et Santé des Populations, Université Paris-Saclay, Université de Versailles Saint-Quentin-en-Yvelines, Université Paris-Saclay, Inserm, High-Dimensional Biostatistics for Drug Safety and Genomics, Villejuif, France; ^3^University Committee of Resources for Research in Health (CURRS), University of Reims, Champagne-Ardenne, Reims, France; ^4^Pole Territorial Santé Publique et Performance, Hôpitaux Champagne Sud, Troyes, France; ^5^General Practice Department, University of Reims Champagne-Ardenne, Reims, France; ^6^Clinical Epidemiology and Clinical Trials Unit, Biostatistics and Bioinformatics (DIM), Centre d'Investigation Clinique 1432, Clinical Investigation Center, Dijon University Hospital, Dijon, France; ^7^Inserm, Centre de recherche en Epidémiologie et Santé des Populations, Université Paris-Saclay, Université de Versailles Saint-Quentin-en-Yvelines, Villejuif, France

**Keywords:** pay-for-performance (P4P), general practitioner, quality of care, P4P, France

## Abstract

**Background:**

Pay-for-performance (P4P) models are intended to promote quality of care in both hospitals and primary care settings. They are considered as a means of changing medical practices, particularly in primary care.

**Objectives:**

The first objective of this study was to assess how performance indicators changed over time, measured through “Remuneration on Public Health Objectives” (ROSP) scores, between 2017 and 2020 in a large French region (Grand Est region), and to compare this evolution in the rural vs. urban areas of the region. The second objective was to focus on the area with the least improvement in ROSP scores and to investigate whether the scores and the available sociodemographic characteristics of the area were associated.

**Methods:**

First, we measured the evolution over time of P4P indicators (i.e., ROSP scores) obtained from the regional health insurance system, for GP practices in the Grand Est region between 2017 and 2020. We then compared the scores between the Aube Department and the rest of the region (urban areas). To address the second objective, we focused on the area found to have the least improvement in indicators to investigate whether there was a relationship between ROSP score and sociodemographic characteristics.

**Results:**

More than 40,000 scores were collected. We observed an overall improvement in scores over the study period. The urban area (Grand Est region minus the Aube) scored better than the rural area (Aube) for chronic disease management [median 0.91 (0.84–0.95) vs. 0.90(0.79–0.94), *p* < 0.001] and prevention [median 0.36 (0.22–0.45) vs. 0.33 (0.17–0.43), *p* < 0.001], but not for efficiency, where the rural area (Aube) performed better [median 0.67(0.56–0.74) vs. 0.69 (0.57–0.75 in the rest of the Grand Est region, *p* = 0.004]. In the rural area, we found no significant association between ROSP scores and sociodemographic characteristics, except for extreme rurality in some sub-areas.

**Conclusions:**

At the regional level, the overall improvement in scores observed between 2017 and 2020 suggests that the implementation of ROSP indicators have improved the quality of care, particularly in urban areas. These results also suggest that efforts should be focused on rural areas, which already had the lowest scores at the start of the P4P program.

## 1. Introduction

Pay for performance (P4P) models are used to improve the quality of care through economic incentives that are based on the achievement of quality indicators. These models are now widely used in the form of mixed payments (fee-for-service and contracting) and represent the first step in shifting from fee-for-service to capitation-based models. When primary care is predominantly funded on a fee-for-service basis, introducing a P4P model may help to change practices and promote prevention. Indeed, in the P4P model, payment is based on the number of patients being treated (for example, for chronic conditions) rather than on the number of individual procedures. When payment is on a fee-for-service basis, it can lead to artificial “inflation” of the number of procedures (1). With fee-for-service models, there is a propensity to give precedence to quantity at the cost of quality of care, contrary to capitation-based models, which favor quality (2).

P4P programs have been part of numerous experiments in both the hospital and ambulatory care sectors. In 2004, the United Kingdom (UK) was one of the first countries to introduce this type of model with the Quality and Outcomes Framework, which was designed to change medical practices through the use of performance indicators. Nevertheless, some evaluation studies indicated that performance indicators may not be directly beneficial in the hospital sector (3–5) or in primary care (6, 7). Many parameters, such as the type of health insurance system and whether patients are seen in ambulatory versus hospital-based settings, may interfere with the results of these P4P programs. While interpreting and evaluating the effects of financial incentives is not a straightforward task (8), this innovative financing approach is a lever for improving practices in various care settings.

Achieving the objectives set by health authorities can be challenging for healthcare professionals, and physicians in disadvantaged areas often have greater difficulty achieving P4P program goals (9), as has recently been observed in a study from the United States (10). However, in areas with lower baseline performance indicators, P4P models may be particularly useful since there is room for significant improvement (11).

In France, an experimental measure based on voluntary participation, the Contract for Improvement of Individual Practice (Contrat d'Amélioration des Pratiques Individuelles—CAPI), was launched in 2008 to introduce payment by capitation into the remuneration of general practitioners (GP). In 2011, this measure was extended and became Remuneration based on Public Health Objectives (Rémunération sur Objectifs de Santé Publique—ROSP). ROSP applies to GPs as well as to certain specialists and is regularly updated. Currently, it includes 29 clinical indicators for GPs caring for adult patients. This P4P approach rewards all GPs by providing additional payments based on the level of achievement of ROSP indicators, as assessed by quality indicators. The list of indicators is known, so GPs can consult the expected performance criteria for this additional source of income. However, the implementation of ROSPs has been relatively slow: the first payments to GPs were made in 2013, and the number of indicators was expanded in 2016. The first evaluation of the effects of ROSP on physician remuneration took place in 2018. This system is based on a contract between GPs and the national health insurance system, which sets rates of payment according to the level of achievement of each indicator, measured by the scores obtained (National Health Insurance, 2022. La Rosp du médecin traitant de l'adulte. https://www.ameli.fr/medecin/exercice-liberal/remuneration/remuneration-objectifs/medecin-traitant-adulte=). A previous study reported wide variability in obtained scores, which was attributed to the type of physician and their geographical location (12). In this regard, remoteness is a known limiting factor for the use of primary care (in general or specialized medicine) (13–17). We hypothesized that this limitation could negatively impact the quality of care and may be reflected by lower ROSP scores.

The first objective of this study was therefore to measure the evolution in performance indicators between 2017 and 2020, as measured by ROSP scores, in a large French region (Grand Est region) and to compare the changes in scores between the different areas of the region (rural and urban areas). The second objective of the study was to focus on the area with the least improvement in ROSP scores to investigate whether there was an association between the scores and available sociodemographic characteristics.

## 2. Methods

We performed a retrospective cohort study using data obtained from the Regional Health Insurance System. These routine reimbursement data include payments to physicians based on ROSP scores. ROSP scores are calculated for each individual GP, and they measure the level of achievement for each indicator. A detailed description of the calculation method is given in the Supplementary Figure 1 and Supplementary Table 1. We constructed our analyses in line with the two objectives. First, we sought to investigate whether there was an improvement over time following the implementation of P4P in the region for which we had data (Grand Est region). Then, if an improvement was observed, we compared the course of ROSP scores between the different areas of this region (rural: Aube department, and urban: the rest of the Grand-Est region). To address the second objective, we then focused on the area with the least improvement in ROSP scores in order to assess whether there was an association between the scores and available sociodemographic characteristics. The characteristics we focused on were: population density, potential local accessibility, and sociodemographic category of the area (i.e., urban with poor access to care, city center, rural and unattractive urban area, or rural area).

### 2.1. Primary outcome

We retrieved ROSP scores from 2017 to 2020 for all GPs who were eligible for performance-based payment in the Grand Est, an administrative region in the east of France. Accounting for almost 8% of the French population (5 million inhabitants), the Grand Est region includes five urban areas with more than 250,000 inhabitants each (i.e., Metz, Mulhouse, Nancy, Reims and Strasbourg). The Aube Department, in contrast, is the most rural of the 10 departments that comprise the Grand Est region. We thus compared the Aube department with the rest of the Grand Est region (excluding the Aube). Apart from the difference in population density, the two areas are very similar, which simplifies the comparison. More specifically, the Aube Department and the Grand Est region as a whole are alike in terms of sociodemographic characteristics such as population change, proportion of vacant housing, proportion of taxed households, and employment rate among 15–64 year olds. However, the share of agriculture is lower in the rest of the Grand Est region (7.4 vs. 15.5% in 2019), and the population density is very different (96.7 inhabitants/km2 in the Grand Est region vs. 51.7 in the Aube department) (INSEE: statistics and studies—Comparator of territory-region of the Grand Est.c2022 Available from: https://www.insee.fr/fr/statistiques/1405599?geo=REG-44+DEP-08+DEP-10+DEP-51+DEP-52+DEP-54+DEP-55+DEP-57+DEP-67+DEP-68+DEP-88). We therefore hypothesized that the comparison of these two areas (i.e., the Aube department vs. the rest of the Grand Est region) would highlight differences in GPs' practices in rural and urban areas.

The measurement of ROSP indicators was an existing metric that concerns all GPs and that could be used in this framework of this study. The ROSP indicators are defined by the national health insurance system, and are applicable to three areas of GPs' clinical practice, namely: monitoring of chronic diseases, prevention measures, and efficiency of care. For the national health insurance system, these ROSP indicators are used to measure the quality of care and medical practices. For the majority of the indicators, the aim is to exceed the threshold value defined for each indicator. There are 29 indicators, for a total of 940 points. Each point has a monetary value of 7 euros. In addition to reaching the target rates set by the health insurance system, GPs must treat a minimum number of patients in order to be eligible for financial rewards *via* the ROSP system. The ROSP scores in the Aube department and the rest of the Grand Est region were compared overall (Supplementary Table 1).

Concerning the Iatrogenesis and Antibiotic-use indicators, the objectives for GPs involve limitation or reduction, i.e., lower scores are better. For example, for the indicator Percentage of patients aged >75 years old who do not have documented long-term psychiatric disorders and who have ≥2 prescribed psychotropic drugs (excluding anxiolytics) is in the Iatrogenesis category. The intermediate objective was to limit this prescription rate to 10% of patients meeting the definition, with an ultimate target of 3% or fewer. Only four indicators require that each GP connects individually to the health insurance website to declare their activity in view of ROSP indicator calculation (Ameli.fr). For all other indicators, the GP is not required to provide any information. The health insurance system computes the indicators automatically and calculates the total financial reward to be allocated to each physician.

### 2.2. Definitions for classification

For the second part of the study, to take into account potential geographical, social and healthcare differences, we classified the Aube department using three methods: (i) the French Office of National Statistics population density grid classification for municipalities was used to classify municipalities as either “high population density zones” (densely populated and intermediate density), or “low population density zones” (sparsely populated, or very sparsely populated); (ii) the local potential accessibility (LPA) score, which is a measure of the supply of and demand for GPs that takes into account volume of activity, and service use rates differentiated by population age structure. LPA was categorized as “high-accessibility” (if the values were above the median value of the LPA score) or “low-accessibility” (if values were below the median LPA score); and (iii) the Institute for Research and Documentation in the Economics of Health (IRDES) social and health classification (in 6 classes), including supply and demand for healthcare and the attractiveness of the area (details given in Supplementary Table 2).

### 2.3. Statistical analysis

Due to the asymmetric nature of the data collected and the presence of outliers, we used median values for our statistical analyses. Wilcoxon tests were used to compare the three ROSP categories, and the sub-categories for the three classifications described above, in the Grand Est region and Aube department between 2017 and 2020. We also assessed the trends in ROSP scores over the four study years using the Kruskal-Wallis test. A *p*-value < 0.05 was considered statistically significant. All analyses were performed using SAS software version 9.4 (SAS Institute Inc., Cary, NC).

### 2.4. Ethical considerations

This study was conducted in accordance with national laws regarding epidemiological research and data protection. Since this study was entirely retrospective and observational, and relied solely on anonymous data (no personal data), neither ethical approval nor written consent were required.

## 4. Results

We compared 1,919 ROSP scores from the Aube department to 39,017 ROSP scores from the remainder of the Grand Est region. All of the scores were generated between 2017 and 2020.

Between 2017 and 2020, the results tended to improve throughout the Grand Est region, including in the Aube department (Table 1). There was an improvement in Chronic disease follow-up, except for cardiovascular risk (rate variation: −1.96% for Aube, −5.36% for Grand Est). Concerning Prevention, the Cancer indicator decreased between 2017 and 2020 for the Aube Department, but was stable for the Grand Est region. The results for Iatrogenesis and Antibiotic use also improved (indicated by a decreased ROSP score) for the Aube and the Grand Est. For Efficiency, ROSP scores were higher for the Aube compared to Grand Est, and there was a greater increase between 2017 and 2020 for the Aube (rate increase: 27.27% for Aube, 25.45% for Grand Est). ROSP scores for Influenza were null for the Aube and the Grand Est in 2020.

**Table 1:** Changes in ROSP scores between 2017 and 2020 in the Grand–Est region, between Aube and the rest of the Grand Est region.

	**2017**		**2018**		**2019**		**2020**		**Rate of evolution**		
	**Aube**	**GE** ^a^	**Aube**	**GE** ^a^	**Aube**	**GE** ^a^	**Aube**	**GE** ^a^	**Aube**	**GE** ^a^	**Delta**
	**Median (IQR)**	**Median (IQR)**	**Median (IQR)**	**Median (IQR)**	**Median (IQR)**	**Median (IQR)**	**Median (IQR)**	**Median (IQR)**			
**Chronic disease type**	**0.87 (0.71–0.91)**	**0.86 (0.80–0.91)**	**0.90 (0.83–0.94)**	**0.91 (0.86–0.94)**	**0.93 (0.81–0.95)**	**0.93 (0.89–0.96)**	**0.91 (0.78–0.94)**	**0.92 (0.87–0.95)**	**4.60**	**6.98**	**−2.38**
Diabetes	0.56 (0.47–0.67)	0.57 (0.47–0.67)	0.64 (0.53–0.72)	0.62 (0.51–0.71)	0.64 (0.53–0.71)	0.64 (0.52–0.72)	0.62 (0.50–0.70)	0.62 (0.50–0.71)	10.71	8.77	1.94
High blood pressure	0.90 (0.87–0.92)	0.89 (0.85–0.91)	0.94 (0.91–0.96)	0.94 (0.91–0.96)	0.96 (0.94–0.98)	0.96 (0.95–0.98)	0.94 (0.92–0.96)	0.95 (0.93–0.97)	4.44	6.74	−2.3
Cardiovascular risk	0.51 (0.43–0.60)	0.56 (0.48–0.65)	0.53 (0.43–0.61)	0.57 (0.50–0.66)	0.51 (0.43–0.60)	0.55 (0.47–0.64)	0.50 (0.40–0.59)	0.53 (0.44–0.62)	−1.96	−5.36	3.4
**Prevention**	**0.36 (0.20–0.44)**	**0.40 (0.25–0.48)**	**0.38 (0.20–0.45)**	**0.39 (0.25–0.47)**	**0.36 (0.20–0.44)**	**0.38 (0.24–0.46)**	**0.26 (0.12–0.33)**	**0.29 (0.15–0.36)**	**−27.78**	**−27.50**	**−0.28**
Influenza	0.87 (0.71–0.91)	0.50 (0.36–0.59)	0.50 (0.40–0.60)	0.51 (0.39–0.62)	0.52 (0.42–0.63)	0.52 (0.40–0.64)	0.00 (0.00–0.00)	0.00 (0.00–0.00)	−100.00	−100.00	0
Cancer	0.56 (0.47–0.67)	0.48 (0.33–0.55)	0.42 (0.29–0.50)	0.47 (0.33–0.54)	0.43 (0.29–0.50)	0.47 (0.33–0.54)	0.44 (0.31–0.53)	0.48 (0.33–0.56)	7.32	0.00	7.32
Iatrogenesis	0.90 (0.87–0.92)	0.12 (0.00–0.17)	0.12 (0.00–0.17)	0.12 (0.00–0.17)	0.12 (0.00–0.16)	0.11 (0.00–0.16)	0.11 (0.00–0.16)	0.11 (0.00–0.16)	−15.38	−8.33	−7.05
Antibiotic–use	0.51 (0.43–0.60)	0.26 (0.00–0.46)	0.24 (0.00–0.41)	0.25 (0.00–0.44)	0.16 (0.00–0.37)	0.22 (0.00–0.40)	0.14 (0.00–0.34)	0.18 (0.00–0.35)	−41.67	−30.77	−10.9
**Efficiency**	**0.36 (0.20–0.44)**	**0.55 (0.50–0.63)**	**0.69 (0.63–0.75)**	**0.68 (0.63–0.74)**	**0.72 (0.67–0.77)**	**0.71 (0.65–0.76)**	**0.70 (0.65–0.65)**	**0.69 (0.64–0.75)**	**27.27**	**25.45**	**1.82**
Prescription of generics	0.54 (0.50–0.60)	0.54 (0.49–0.60)	0.69 (0.62–0.73)	0.68 (0.62–0.72)	0.71 (0.66–0.76)	0.70 (0.65–0.75)	0.69 (0.64–0.74)	0.69 (0.63–0.73)	27.78	27.78	0
Prescription of biosimilars	0.00 (0.00–0.04)	0.00 (0.00–0.03)	0.08 (0.02–0.17)	0.03 (0.00–0.14)	0.13 (0.06–0.27)	0.09 (0.00–0.24)	0.25 (0.13–0.44)	0.20 (0.04–0.41)	0.17	0.17	0
Efficiency of prescriptions	0.93 (0.80–0.97)	0.91 (0.81–0.96)	0.93 (0.85–0.98)	0.93 (0.85–0.97)	0.95 (0.85–1.00)	0.93 (0.86–0.98)	0.94 (0.86–1.00)	0.93 (0.86–0.98)	1.08	2.20	−1.12

IQR; interquartile range. The delta corresponds to the difference between the evolution rate of rural and urban area. A positive result indicates a better evolution for the rural area while a negative result indicates worse result for the rural area.

^a^GE: Grand Est.

Overall ROSP scores between 2017 and 2020 were compared between the Aube department and the rest of the Grand Est region (excluding the Aube) (Table 2). For indicators relating to chronic diseases, prevention and efficiency of care, while the results were significantly different, the differences were numerically small. Within each category, there were more marked differences between the Aube and the Grand Est for certain sub-criteria, such as the ROSP indicators for cardiovascular risk (median value Aube = 0.51 vs. Grand Est = 0.56, *p* < 0.001), antibiotic prescription (median Aube = 0.19 vs. Grand Est = 0.23, *p* < 0.001) and prescription of biosimilars (median Aube = 0.10 vs. median Grand Est = 0.05, *p* < 0.001).

**Table 2:** Comparison of remuneration based on public health objectives (ROSP) scores in the Aube department vs. the rest of the Grand-Est Region between 2017 and 2020.

	**Aube department *N* = 1,919**	**Grand Est region** ***N* = 39,017**	**p-value**
	**Median (IQR)**	**Median (IQR)**	**p-value**
**Chronic disease**	**0.90 (0.79–0.94)**	**0.91 (0.84–0.95)**	**< 0.001**
Diabetes	0.61 (0.50–0.69)	0.61 (0.50–0.70)	0.492
High blood pressure	0.94 (0.91–0.97)	0.94 (0.91–0.97)	0.333
Cardiovascular risk	0.51 (0.43–0.60)	0.56 (0.47–0.64)	< 0.001
**Prevention**	**0.33 (0.17–0.43)**	**0.36 (0.22–0.45)**	**< 0.001**
Influenza	0.45 (0.00–0.56)	0.44 (0.00–0.57)	0.923
Cancer	0.43 (0.30–0.50)	0.48 (0.33–0.55)	< 0.001
Iatrogenesis	0.12 (0.00–0.17)	0.11 (0.00–0.17)	< 0.001
Antibiotic–use	0.19 (0.00–0.39)	0.23 (0.00–0.41)	< 0.001
**Efficiency**	**0.69 (0.57–0.75)**	**0.67 (0.56–0.74)**	**0.004**
Prescription of generics	0.68 (0.55–0.73)	0.67 (0.56–0.73)	0.108
Prescription of biosimilars	0.10 (0.00–0.24)	0.05 (0.00–0.20)	< 0.001
Efficiency of prescriptions	0.94 (0.85–1.00)	0.93 (0.84–0.97)	< 0.001

IQR. interquartile range.

In terms of prevention, cancer prevention was significantly worse in the Aube department, with a difference of 0.05 points (InterQuartile Range (IQR) Aube = 0.43 vs. IQR Grand Est = 0.48, *p* < 0.001). On the contrary, this department had a better overall efficiency score (IQR Aube = 0.69 vs. IQR Grand Est = 0.67, *p* < 0.004).

Table 3 displays the results according to the population density of the area where the GP's practice was located for GPs in the Aube Department. In terms of chronic disease follow-up, there was no significant difference between high- and low-density areas, except for cardiovascular risk, where low-density zones had better results (median 0.55 vs. 0.50, *p* < 0.001). The opposite was observed for prevention: high-density zones achieved better results for iatrogenesis and prescription of antibiotics (median 0.11 vs. 0.14, *p* < 0.0001, and 0.16 vs. 0.25, *p* < 0.0001, respectively). There was no significant difference in cancer prevention between high- and low- population density areas, but the high-density zones obtained better results for prescription efficiency (median 0.93 vs. 0.94, *p* < 0.0001).

**Table 3:** Comparison of remuneration based on public health objective (ROSP) scores between high- and low-population-density areas in the Aube department between 2017 and 2020.

	**Overall (*n* = 1,919)**	**High–density population (*n* = 1,387)**	**Low–density population (n = 532)**	**p-value**
	**Median (IQR)**	**Median (IQR)**	**Median (IQR)**	
**Chronic disease type**	**0.90 (0.79–0.94)**	**0.90 (0.78–0.94)**	**0.91 (0.80–0.94)**	**0.95**
Diabetes	0.61 (0.50–0.69)	0.62 (0.50–0.70)	0.60 (0.50–0.69)	0.23
High blood pressure	0.94 (0.91–0.97)	0.94 (0.90–0.97)	0.95 (0.91–0.97)	0.10
Cardiovascular risk	0.51 (0.43–0.60)	0.50 (0.40–0.58)	0.55 (0.48–0.64)	< 0.0001
**Prevention**	**0.33 (0.17–0.43)**	**0.33 (0.15–0.43)**	**0.35 (0.22–0.42)**	**0.002**
Influenza	0.45 (0.00–0.56)	0.45 (0.00–0.57)	0.46 (0.00–0.55)	0.83
Cancer	0.43 (0.30–0.50)	0.43 (0.22–0.50)	0.42 (0.35–0.49)	0.09
Iatrogenesis	0.12 (0.00–0.17)	0.11 (0.00–0.16)	0.14 (0.08–0.17)	< 0.0001
Antibiotic-use	0.19 (0.00–0.39)	0.16 (0.00–0.38)	0.25 (0.00–0.40)	0.0001
**Efficiency**	**0.69 (0.57–0.75)**	**0.68 (0.56–0.76)**	**0.69 (0.58–0.74)**	**0.71**
Prescription of generics	0.68 (0.55–0.73)	0.67 (0.55–0.74)	0.69 (0.57–0.73)	0.41
Prescription of biosimilars	0.10 (0.00–0.24)	0.10 (0.01–0.24)	0.09 (0.00–0.23)	0.56
Efficiency of prescriptions	0.94 (0.85–1.00)	0.94 (0.85–1.00)	0.93 (0.82–0.97)	< 0.0001

IQR; interquartile range. Note: Classifications were according to the national institute of statistics and economic studies (INSEE) data for population density.

ROSP scores according to high vs. low potential accessibility in the Aube Department are presented in Table 4. The overall score for chronic disease follow-up was lower in low-accessibility zones than in high-accessibility zones (median 0.90 vs. 0.91, *p* < 0.02). Conversely, for cardiovascular risk, low-accessibility zones had higher scores (median 0.53 vs. 0.50, *p* < 0.0001).

**Table 4:** Comparison of ROSP scores in the Aube department between zones with high (>median) vs. low (< median) local potential accessibility (LPA) score from 2017 to 2020.

	**Overall (*n* = 1,919)**	**High-accessibility zones (*n* = 960)**	**Low-accessibility zones (*n* = 959)**	**p-value**
	**Median (IQR)**	**Median (IQR)**	**Median (IQR)**	
**Chronic disease**	**0.90 (0.79–0.94)**	**0.91 (0.80–0.95)**	**0.90 (0.78–0.93)**	**0.02**
Diabetes	0.61 (0.50–0.69)	0.61 (0.50–0.69)	0.61 (0.50–0.70)	0.59
High blood pressure	0.94 (0.91–0.97)	0.94 (0.91–0.97)	0.94 (0.91–0.96)	0.09
Cardiovascular risk	0.51 (0.43–0.60)	0.50 (0.39–0.56)	0.53 (0.45–0.62)	< 0.001
**Prevention**	**0.33 (0.17–0.43)**	**0.33 (0.16–0.44)**	**0.33 (0.19–0.42)**	**0.47**
Influenza	0.45 (0.00–0.56)	0.47 (0.00–0.59)	0.44 (0.00–0.55)	0.03
Cancer	0.43 (0.30–0.50)	0.43 (0.23–0.51)	0.41 (0.32–0.50)	0.84
Iatrogenesis	0.12 (0.00–0.17)	0.10 (0.00–0.15)	0.13 (0.06–0.17)	< 0.001
Antibiotic-use	0.19 (0.00–0.39)	0.12 (0.00–0.36)	0.24 (0.00–0.40)	< 0.001
**Efficiency**	**0.69 (0.57–0.75)**	**0.68 (0.56–0.79)**	**0.69 (0.57–0.74)**	**0.59**
Prescription of generics	0.68 (0.55–0.73)	0.67 (0.55–0.75)	0.68 (0.56–0.73)	0.63
Prescription of biosimilars	0.10 (0.00–0.24)	0.10 (0.004–0.23)	0.10(0.002–0.24)	0.71
Efficiency of prescriptions	0.94 (0.85–1.00)	0.95 (0.89–1.00)	0.93 (0.81–0.97)	< 0.0001

IQR, interquartile range.

Regarding prevention, the high-accessibility zones seemed to perform better for the risk of iatrogenesis and prescription of antibiotics (median 0.13 vs. 0.10, *p* < 0.0001 and median 0.24 vs. 0.12, *p* < 0.0001, respectively). The high-accessibility zones also had a better score for prescription efficiency (median 0.93 vs. 0.95, *p* < 0.001).

Table 5 shows a comparison within the Aube Department according to categories of health and social criteria from the IRDES (French Institute for Research and Documentation in the Economics of Health). When significant differences were observed, lower ROSP scores were associated with the socio-economic category (Rural outskirts with low attractiveness and vulnerable populations) for the following indicators: Chronic Disease (median 0.89 vs. 0.9 (overall), *p* < 0.001), Cancer (median 0.38 vs. 0.42 (overall), *p* = 0.04), and Iatrogenesis [median 0.13 vs. 0.11 (overall), *p* = 0.005].

**Table 5:** Comparison of the territories within the Aube department from 2017 to 2020 according to the IRDES^a^ indicator.

	**Overall (*n* = 1,381)**	**Class 1 (*n* = 221)^1^**	**Class 2 (*n* = 334)^2^**	**Class 4 (*n* = 96)^3^**	**Class 5 (*n* = 730)^4^**	**p^†^**
	**Median (IQR** ^b^ **)**	**Median (IQR** ^b^ **)**	**Median (IQR** ^b^ **)**	**Median (IQR** ^b^ **)**	**Median (IQR** ^b^ **)**	
**Chronic disease**	**0.90 (0.78–0.94)**	**0.89 (0.60–0.93)**	**0.89 (0.70–0.93)**	**0.91 (0.67–0.97)**	**0.91 (0.81–0.96)**	**0.0002**
Diabetes	0.60 (0.50–0.67)	0.59 (0.49–0.67)	0.61 (0.49–0.68)	0.60 (0.40–0.67)	0.61 (0.50–0.68)	0.41
High blood pressure	0.94 (0.91–0.97)	0.94 (0.90–0.96)	0.94 (0.90–0.96)	0.95 (0.91–1.00)	0.94 (0.92–0.97)	0.03
Cardiovascular risk	0.50 (0.40–0.59)	0.46 (0.21–0.54)	0.52 (0.44–0.64)	0.50 (0.10–0.59)	0.50 (0.40–0.57)	< 0.0001
**Prevention**	**0.33 (0.15–0.43)**	**0.32 (0.12–0.41)**	**0.32 (0.17–0.42)**	**0.27 (0.07–0.40)**	**0.33 (0.17–0.45)**	**0.15**
Influenza	0.45 (0.00–0.57)	0.44 (0.00–0.56)	0.43 (0.00–0.54)	0.33 (0.00–0.57)	0.47 (0.00–0.62)	0.04
Cancer	0.42 (0.22–0.50)	0.43 (0.15–0.50)	0.38 (0.23–0.48)	0.43 (0.14–0.51)	0.43 (0.23–0.51)	0.04
Iatrogenics	0.11 (0.00–0.16)	0.11 (0.00–0.15)	0.13 (0.02–0.17)	0.07 (0.00–0.17)	0.10 (0.00–0.16)	0.005
Antibiotics	0.15 (0.00–0.38)	0.14 (0.00–0.35)	0.21 (0.00–0.39)	0.12 (0.00–0.33)	0.12 (0.00–0.39)	0.08
**Efficiency**	**0.68 (0.56–0.76)**	**0.70 (0.58–0.82)**	**0.69 (0.56–0.73)**	**0.67 (0.50–0.78)**	**0.68 (0.55–0.79)**	**0.11**
Prescription of generics	0.67 (0.54–0.74)	0.69 (0.56–0.77)	0.68 (0.54–0.71)	0.67 (0.50–0.76)	0.67 (0.54–0.75)	0.07
Prescription of biosimilars	0.10 (0.01–0.22)	0.12 (0.03–0.23)	0.08 (0.01–0.18)	0.14 (0.08–0.36)	0.09 (0.00–0.23)	0.19
Efficiency of prescriptions	0.94 (0.85–1.00)	0.94 (0.90–1.00)	0.92 (0.83–0.97)	0.91 (0.67–0.95)	0.95 (0.85–1.00)	< 0.0001

^a^IRDES, Institute for Research and Documentation in the Economics of Health.

^b^IQR, interquartile range.

^†^Comparison of the 4 categories

1: Class 1—Urban areas with poor accessibility to health care.

2: Class 2—Rural outskirts with low attractiveness and vulnerable populations.

3: Class 4—Underprivileged urban or rural areas with low socio–economic indicators and poor access to health care.

4: Class 5—City centers with socio–economic diversity, with high availability of health care.

## 5. Discussion

In our analysis of the temporal trends in ROSP scores from 2017 to 2020, we observed a gradual improvement each year for both the Aube department and the Grand Est region. This result suggests that the implementation ROSP has a positive impact of on quality of care. The increase was particularly marked for the prescription of biosimilars and generic drugs, which is a successful result in view of current health policies that aim to restrict health expenditures. This finding has also been described in the literature (18). Our results show that the urban area (Grand Est region) had better scores for chronic disease management and prevention, whereas the rural area (Aube) performed better for efficiency. However, the literature does not always show positive effects for these quality of care incentives. A recent study showed that P4P scores were inconsistently associated with quality improvement, which raises questions about the usefulness of the incentives (19).

In the Aube Department, it is worth underlining that overall ROSP scores were similar regardless of the population density (high-density vs. low-density). This shows that GPs can achieve similar quality of care outcomes within a rural area that is supposedly heterogeneous in terms of population density. However, scores in the Prevention category were worse in low-population-density areas for cancer screening, iatrogenesis and antibiotic use.

Again for the Aube department, the Chronic Disease indicator scored worse in areas with a lower potential accessibility score, although the difference in scores was very small. This result should be weighed against the fact that scores were higher for the Cardiovascular risk subcategory in areas with a low LPA score. Our results therefore only partly corroborate those of the literature, where it has been reported that GP activity differs in the city and in the countryside, with those practicing in rural areas tending to manage more patients with chronic diseases and to perform fewer preventive acts (20). The IRDES classification provides additional results, showing that urban areas with poor access to care had the lowest cardiovascular scores. However, the most rural areas within the Aube department had lower scores on the Chronic Disease, Cancer, and Iatrogenesis indicators, again highlighting significant differences within our rural study area. The prevention and efficiency scores did not differ according to the IRDES classification.

Our results can be at least partially explained by established biases of P4P programs in private practice. It is known that patients for whom P4P goals are more achievable receive more care (21). In addition, difficulty accessing specialists, such as cardiologists, may lead primary care physicians to over-medicate patients with certain conditions, and this would indirectly affect the ROSP scores compared to other regions. In this case, the indicators reflect more the difference in patients treated between urban and rural areas than the difference in practices related to the professionals themselves. The poorer results obtained in the areas in the Aube department with low potential accessibility could reflect shorter consultation times due to an increased burden of work for health professionals, especially GPs (22). The lack of time to explain the reasons for antibiotic abstention and to offer additional follow-up consultations could explain the over-prescription of antibiotics.

Overall, our study provides original results by seeking to compare practices between urban and rural areas and within a rural area based on P4P indicators. This investigation was made possible by access to this novel database. Ultimately, our work could be used to develop specific indicators to monitor the quality of care provided, and to provide insights into how we can best adapt the resources available to health professionals in rural areas.

This study has some limitations. Firstly, there is potential for selection bias because our statistics only include GPs who are registered for the P4P system. Although this represents the majority of physicians, it is important to note that their practices may differ from those of GPs who were not registered. We also know that GP have specific motivations for settling in urban vs. rural areas. While the majority of GPs choose to set up their practice in the region where they did their residency training, the criteria for choosing a more or less urban area are predominantly related to the dynamics of supply of care, demand for care and living conditions in the area (23–25). Furthermore, it is not possible to fully assess the magnitude of the effect of ROSP scores on population health without first considering the case mix. The difficulty of assessing the overall impact is compounded by the frequent changes to the indicators, meaning that any assessment of the data and their relationship to patient health is necessarily limited to a short period of time. However, based on the trends we observed for the criteria studied, we can suggest that this limit seems well under control. We obtained results for only one large French region (Grand Est). However, this region has many points in common with the other French regions in terms of healthcare delivery. The design of the article did not enable direct assessment of the impact of the intervention through a comparison of the “here-vs-elsewhere” type, since we were not comparing two areas (i.e., one receiving the intervention and one not). The comparison of intervention vs. non-intervention areas was not possible because, subsequent to the Ministry of Health decision, P4P was implemented on a national level in a uniform manner. All regions in France implemented P4P at the same time. It was also not possible to conduct a before-and-after evaluation, because the available data did not include information at T0, before the intervention began.

It is common practice to evaluate public health policies with a time lag of several years in order to be more objective about the real impact of reforms, as it always takes time for practices to adapt, especially for GPs. Our study had 6 years of hindsight, which seemed reasonable to us. Our data therefore provide information on the evolution of ROSP scores (P4P indicators) during the implementation of P4P in a large French region, allowing us to judge whether a benefit could be expected from this implementation. We also sought to investigate whether the changes over time were different in rural vs. urban areas, bearing in mind that the entire region started P4P at the same time. It would have been very interesting to be able to compare the 2 areas according to population density, LPA, and IRDES classification. Unfortunately, we were unable to obtain sufficiently exhaustive data for the rest of the Grand Est region to ensure the validity of the comparison. Further studies are therefore needed to expand on this comparison and to be able to conclude on the impact of P4P.

Practitioners do not always see the introduction of performance-based payment as a positive change, which could contribute to weaker-than-expected improvements in efficiency (25). The implementation of P4P could also lead to over-medicalization as practitioners strive meet the indicator targets (26). Better compliance would likely require greater participation of the healthcare professionals themselves in the co-definition of these indicators. Finally, we cannot rule out a possible classification bias in the definition of groups for our comparisons, which are ultimately based on geographic criteria. However, our results were consistent across the three classification schemes, suggesting a limited effect of classification. It would have been interesting to obtain data on the medical demographics of the other departments of the region to extend our investigation. Future studies could qualitatively investigate the regional profiles of GPs to better understand whether their characteristics explain some of the differences we observed.

## 6. Conclusions

The overall improvement in scores observed between 2017 and 2020 in the Grand Est region suggests that the implementation of ROSP indicators may be useful for improving quality of care in the medium and long term. However, the comparison of ROSP scores in rural and urban areas revealed certain differences, with urban areas doing better overall. When we focused on the rural area (Aube department), our data showed that the scores varied little according to the density of the sub-areas. However, significant differences were observed for some of the social criteria scores, showing lower ROSP scores for the extreme rurality (“Unattractive rural periphery”) of an area. These results suggest that efforts should be concentrated on rural areas, which already had the lowest scores when P4P was first implemented, and which have seen fewer P4P-related benefits than their more urban neighbors.

## Data availability statement

The data analyzed in this study is subject to the following licenses/restrictions: These data are provided by the primary health insurance fund, especially for the purposes of this study. They are therefore not available to the public. Requests to access these datasets should be directed to SS: stephane.sanchez@hcs-sante.fr.

## Author contributions

LP and AO-H were involved in the conception and design of the study. SS and CQ were the coordinator of the study. LP and AO-H were responsible for the data collection. M-AS wrote the first draft. LB was in charge of the analysis. M-AS and CQ were involved in the interpretation and critically reviewed the first draft. All authors approved the final version and accept responsibility for the paper as published.

## References

[1] Khullar D Schpero WL Bond AM Qian Y Casalino LP. Association between patient social risk and physician performance scores in the first year of the merit-based incentive payment system. JAMA. (2020) 324:975–83. 10.1001/jama.2020.13129 32897345 PMC7489811

[2] Chee TT Ryan AM Wasfy JH Borden WB. Current State of value-based purchasing programs. Circulation. (2016) 133:2197-205. 10.1161/CIRCULATIONAHA.115.010268 27245648 PMC5378385

[3] Campbell SM Reeves D Kontopantelis E Sibbald B Roland M. Effects of pay for performance on the quality of primary care in England. N Engl J Med. (2009) 361:368–78. 10.1056/NEJMsa0807651 19625717

[4] Milstein R Schreyoegg J. Pay for performance in the inpatient sector: a review of 34 P4P programs in 14 OECD countries. Health Policy. (2016) 120:1125–40. 10.1016/j.healthpol.2016.08.009 27745916

[5] Emmert M Eijkenaar F Kemter H Esslinger AS Schöffski O. Economic evaluation of pay-for-performance in health care: a systematic review. Eur J Health Econ. (2012) 13:755–67. 10.1007/s10198-011-0329-8 21660562

[6] Pandya A Doran T Zhu J Walker S Arntson E Ryan AM. Modelling the cost-effectiveness of pay-for-performance in primary care in the UK. BMC Med. (2018) 16:135. 10.1186/s12916-018-1126-3 30153827 PMC6114231

[7] Sicsic J Franc C. Impact assessment of a pay-for-performance program on breast cancer screening in France using micro data. Eur J Health Econ. (2017) 18:609–21. 10.1007/s10198-016-0813-2 27329654

[8] Counte MA Howard SW Chang L Aaronson W. Global advances in value-based payment and their implications for global health management education, development, and practice. Front Public Health. (2018) 6:379. 10.3389/fpubh.2018.00379 30713838 PMC6345717

[9] Doran T Fullwood C Gravelle H Reeves D Kontopantelis E Hiroeh U. Pay-for-performance programs in family practices in the United Kingdom. N Engl J Med. (2006) 355:375–84. 10.1056/NEJMsa055505 16870916

[10] Khullar D Bond AM Qian Y O'Donnell E Gans DN Casalino LP. Physician practice leaders' perceptions of medicare's merit-based incentive payment system (MIPS). J Gen Intern Med. (2021) 36:3752–8. 10.1007/s11606-021-06758-w 33835310 PMC8034038

[11] Mendelson A Kondo K Damberg C Low A Motúapuaka M Freeman M. The effects of pay-for-performance programs on health, health care use, and processes of care: a systematic review. Ann Intern Med. (2017) 166:341–53. 10.7326/M16-1881 28114600

[12] Chho C. Les Changements Comportementaux Induits Par La Rémunération sur Objectifs de Santé Publique (ROSP). Paris, France: Université de Paris XI, Faculté de médecine (2015).

[13] Gould M Moon G. Problems of providing health care in British Island Communities. Soc Sci Med. (2000) 50:1081–90. 10.1016/s0277-9536(99)00356-1 10714928

[14] Leese GP Ahmed S Newton RW Jung RT Ellingford A Baines P. Use of mobile screening unit for diabetic retinopathy in rural and urban areas. BMJ. (1993) 306:187–9. 10.1136/bmj.306.6871.187 8443485 PMC1676588

[15] Farmer J Lauder W Richards H. Dr John has gone: assessing health professionals' contribution to remote rural community sustainability in the UK. Soc Sci Med. (2003) 57:673–86. 10.1016/s0277-9536(02)00410-0 12821015

[16] Jones AP Bentham G Harrison BD Jarvis D Badminton RM Wareham NJ. Accessibility and health service utilization for asthma in Norfolk, England. J Public Health Med. (1998) 20:312–7. 10.1093/oxfordjournals.pubmed.a024774 9793897

[17] Wakerman J Humphreys J Russell D Guthridge S Bourke L Dunbar T. Remote health workforce turnover and retention: what are the policy and practice priorities? Hum Resour Health. (2019) 17:99. 10.1186/s12960-019-0432-y 31842946 PMC6915930

[18] Michel-Lepage A Ventelou B. The true impact of the French pay-for-performance program on physicians' benzodiazepines prescription behavior. Eur J Health Econ. (2016) 17:723–32. 10.1007/s10198-015-0717-6 26304210

[19] Bond AM Schpero WL Casalino LP Zhang M Khullar D. Association between individual primary care physician merit-based incentive payment system score and measures of process and patient outcomes. JAMA. (2022) 328:2136–46. 10.1001/jama.2022.20619 36472595 PMC9856441

[20] Lurquin B Kellou N Colin C Letrilliart L. Comparison of rural and urban French GPs' activity: a cross-sectional study. Rural Remote Health. (2021) 21:5865. 10.22605/RRH5865 34469693

[21] Oxholm AS Di Guida S Gyrd-Hansen D. Allocation of health care under pay for performance: winners and losers. Soc Sci Med. (2021) 278:113939. 10.1016/j.socscimed.2021.113939 33962321

[22] Rabinowitz HK Paynter NPMSJAMA. The rural vs urban practice decision. JAMA. (2002) 287:113. 10.1001/jama.287.1.113-JMS0102-7-1 11754723

[23] Kent M Verstappen AC Wilkinson T Poole P. Keeping them interested: a national study of factors that change medical student interest in working rurally. Rural Remote Health. (2018) 18:4872. 10.22605/RRH4872 30293435

[24] Henry JA Edwards BJ Crotty B. Why do medical graduates choose rural careers? Rural Remote Health. (2009) 9:1083. 10.22605/RRH1083 19257797

[25] Giancotti M Mauro M Rania F. Exploring the effectiveness of a P4P scheme from the perspective of Italian general practitioners: a replication study. Int J Health Plann Manage. (2022) 37:1526–44. 10.1002/hpm.3417 35067968

[26] Zwaagstra Salvado E van Elten HJ van Raaij EM. The linkages between reimbursement and prevention: a mixed-methods approach. Front Public Health. (2021) 9:750122. 10.3389/fpubh.2021.750122 34778183 PMC8578935

