# Supplementary material for: Does the implementation of pay-for-performance indicators improve the quality of healthcare? First results in France
Source: Front Public Health. 2023 Mar 9;11:1063806. doi: 10.3389/fpubh.2023.1063806 (PMC10035788; doi:10.3389/fpubh.2023.1063806)
Supplement: Supplementary file 1 [file Data_Sheet_1.docx]

**Supplementary material:**

**Appendix A: remuneration based on public health objectives scores, Institute for Research and Documentation in the Economics of Health classification**

**
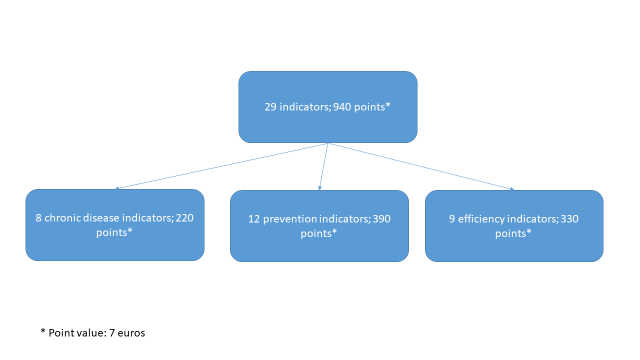
**

Figure 1: ROSP^a^ issues for general practitioners

^a^ROSP: remuneration based on public health objectives

Table 1: Description of ROSP^a^ Criteria and sub-criteria

| Indicator | Criteria | Sub-criteria | Intermediate target | Final target | Threshold | Points |
| --- | --- | --- | --- | --- | --- | --- |
| Chronic Disease | Diabetes | Proportion of patients treated using antidiabetic medication who have had at least two HbA1c tests within the last year. | 74 % | ≥ 92 % | 5 patients | 30 |
|  |  | Proportion of patients treated using antidiabetic medication who have had an ophthalmology consultation, a fundoscopy, or a retinography exam within the last two years. | 60 % | ≥ 76 % | 5 patients | 30 |
|  |  | Proportion of patients aged under 81 years old who are treated using antidiabetic medication and have undergone an annual urine microalbumin test and annual creatinine measurement with calculation of the glomerular filtration rate (GFR). | 21 % | ≥ 59 % | 5 patients | 30 |
|  | High Blood Pressure | Proportion of patients treated using an antihypertensive drug whose proteinuria or microalbuminuria has been tested annually, in addition to creatinuria testing with GFR calculation | 10 % | ≥ 28 % | 5 patients | 30 |
|  | Cardio-vascular risk | Proportion of patients with a history of heart disease or peripheral artery disease treated with statins and an antiplatelet drug, and ACE inhibitors or Angiotensin II receptor blockers. | 38 % | ≥ 56 % | 5 patients | 30 |
|  |  | Proportion of patients receiving a long-term vitamin K antagonist (VKA) treatment who have received at least as many Internal Normalized Ratio (INR) tests as VKA doses over the course of the year (exception if >= 10 INR tests). | 80 % | ≥ 96 % | 5 patients | 30 |

| Prevention | Influenza | Proportion of patients aged 65 years or over who have received the seasonal flu vaccine | 49 % | ≥ 61 % | 5 patients | 20 |
| --- | --- | --- | --- | --- | --- | --- |
|  |  | Proportion of patients aged between 16 and 64 years old with a long-term illness or who have a chronic respiratory condition (asthma, chronic bronchitis, bronchiectasis, bronchial hyperreactivity) who have been targeted by the vaccination campaign and vaccinated | 27 % | ≥ 42 % | 5 patients | 20 |
|  | Cancer | Proportion of female patients between 50 and 74 years old participating in breast cancer screening | 62 % | ≥ 74 % | 5 patients | 40 |
|  |  | Proportion of female patients aged between 25 and 65 years old who have had a cervical smear exam within the last three years | 52 % | ≥ 65 % | 5 patients | 40 |
|  |  | Proportion of patients aged between 50 and 74 years old who have undergone colorectal cancer screening within the last two years | 26 % | ≥ 55 % | 5 patients | 55 |
|  | Iatrogenesis | Proportion of patients >75 years old not categorized as having a long-term psychiatric disease (as defined by the French public health authority) who are being prescribed at least two psychotropic drugs (not including anxiolytics). | 10 % | ≤ 3 % | 5 patients | 35 |
|  |  | Proportion of patients who have commenced a treatment of benzodiazepine hypnotics and whose treatment has lasted more than 4 weeks | 47 % | ≤ 30 % | 5 patients | 35 |
|  |  | Proportion of patients who have commenced a treatment of benzodiazepine hypnotics and whose treatment has lasted more than 12 weeks | 19 % | ≤ 9 % | 5 patients | 35 |
|  | Antibiotic therapy | Number of antibiotic treatments prescribed per 100 patients from 16 to 65 years old, excluding patients with long-term conditions | 45 % | ≤ 20 % | 5 patients | 35 |
|  |  | Proportion of patients treated using antibiotics that are particularly associated with antibiotic resistance (amoxicillin + clavulanic acid; 3rd and 4th generation cephalosporin; fluoroquinolones). | 52 % | ≤ 32 % | 5 patients | 35 |

| Efficiency | Generic drug/biosimilar prescriptions | Number of boxes of statins prescribed from the directory of generic drugs; number of boxes of antihypertension drugs prescribed from the directory of generic drugs | 83 % | ≥ 90 % | 10 boxes | 45 |
| --- | --- | --- | --- | --- | --- | --- |
|  |  | Number of boxes of medication (excluding statins and antihypertension drugs) prescribed from the directory of generic drugs; number of boxes of biosimilars prescribed in comparison with the prescription of the reference product insulin glargine | 4 % | ≥ 40 % | 10 boxes | 30 |
|  | Efficiency of prescriptions | Proportion of patients treated using low dose aspirin in the cohort of patients treated with antiplatelet medication | 83 % | ≥ 92 % | 5 patients | 45 |
|  |  | Proportion of diabetic patients treated with metformin | 76 % | ≥ 90 % | 5 patients | 45 |
|  |  | Patients who have undergone a thyroid stimulating hormone (TSH) test alone as a proportion of all thyroid hormone assays in the previous 15 months | 90 % | ≥ 99 % | 5 patients | 45 |

Data from the National Health Insurance *https://www.ameli.fr/medecin/exercice-liberal/remuneration/remuneration-objectifs/medecin-traitant-adulte*

^a^ROSP: emuneration based on public health objectives

Table 2: IRDES^a^ classification

| Class 1 | Urban areas with poor accessibility to health care |
| --- | --- |
| Class 2 | Rural outskirts with low attractiveness and vulnerable populations |
| Class 3 | Areas popular with retirees and tourists, with high availability of health care |
| Class 4 | Underprivileged urban or rural areas with low socio-economic indicators and poor access to health care |
| Class 5 | City centers with socio-economic diversity, with high availability of health care |
| Class 6 | Wealthy cities and suburbs |

^a^IRDES: Institute for Research and Documentation in the Economics of Health
